# *In vivo* delineation of glioblastoma by targeting tumor-associated macrophages with near-infrared fluorescent silica coated iron oxide nanoparticles in orthotopic xenografts for surgical guidance

**DOI:** 10.1038/s41598-018-29424-4

**Published:** 2018-07-24

**Authors:** Chaedong Lee, Ga Ram Kim, Juhwan Yoon, Sang Eun Kim, Jung Sun Yoo, Yuanzhe Piao

**Affiliations:** 1 Department of Transdisciplinary Studies, Seoul National University, Seoul, Republic of Korea; 2 Department of Nuclear Medicine, Seoul National University Bundang Hospital, Seongnam, Republic of Korea; 3 Advanced Institutes of Convergence Technology, Suwon, Republic of Korea; 4 Department of Health Technology and Informatics, The Hong Kong Polytechnic University, Hong Kong SAR, P. R. China

## Abstract

Glioblastoma multiforme (GBM) is the most aggressive and lethal type of human brain cancer. Surgery is a current gold standard for GBM treatment but the complete surgical resection of GBM is almost impossible due to their diffusive characteristics into surrounded normal brain tissues. There is an urgent need to develop a sensitive imaging tool for accurate delineation of GBM in the operating room to guide surgeons. Here we illustrate the feasibility of using near-infrared fluorescent silica coated iron oxide nanoparticles (NF-SIONs) with high water dispersion capacity and strong fluorescence stability for intraoperative imaging of GBM by targeting tumor-associated macrophages. Abundant macrophage infiltration is a key feature of GBM margins and it is well associated with poor prognosis. We synthesized NF-SIONs of about 37 nm to maximize endocytosis activity for macrophage uptake. The NF-SIONs selectively visualized tumor-associated macrophage populations by *in vitro* live-cell imaging and *in vivo* fluorescence imaging. In the orthotopic GBM xenograft models, the NF-SIONs could successfully penetrate blood-brain barrier and delineated tumor burden specifically. Taken together, this study showcased the potential applications in GBM treatment for improved intraoperative staging and more radical surgery as well as dual modality benefit in order to circumvent previous clinical failure.

## Introduction

Macrophages are essential components of our innate immune systems. Not only they recognize the antigens and neutralize through phagocytosis, but also macrophages regulate the homeostasis of cellular environment. Mature macrophages are differentiated from monocytes through polarization process, then exhibit distinctive expressions as their phenotypes, M1 and M2^1^. While the main function of the M1 macrophages is inducing inflammatory responses and tumor necrosis, the M2 macrophages exert anti-inflammatory responses and promote vascularization, which is highly beneficial for growth and metastasis of cancer cells in tumor microenvironment. Recent studies have shown that tumor-associated macrophages (M2 macrophages) are recognized as important biomarkers in the diagnosis and prognosis of malignant tumors and are thus considered as a potential target for successful tumor therapy^2,3^. Hence, the comprehensive understanding of such tumor-associated macrophages (TAMs) is highly important in successful cancer diagnosis and therapy.

Among the various malignant brain cancers, Glioblastoma multiforme (GBM) is the most frequently encountered disease. Most of GBM patients show extremely low surviving rate (~10%) within 5 years even after the surgical excision and chemo- or radio-therapies^4,5^. Such severe mortality of GBM is significantly related to the population of accumulated tumor-associated macrophages, comprising 30~50% of whole cells in tumor mass and releasing several factors that promote the glioma growth and invasion^6,7^. Furthermore, the residual tumor margin after the surgical resection often recurs the GBM, since the determination of tumor boundary is quite subjective and hardly available only by the naked eye during the operations^8^.

Fluorescence-guided surgery is an emerging technique for improving oncologic intraoperative procedures. The pre-injected fluorescents agents enhance visualization of tumor margins and help to determine the extent of tumor resection in glioma surgery^9–12^. Traditional small molecule based fluorophores have been used to provide intraoperative fluorescence guidance in the tumor region for decades, however, they showed the limited circulation time and lack of diagnostic accuracy and specificity due to the diffusion to adjacent interstitial spaces^13,14^.

To overcome these problems, nanoparticle-based contrast agents were developed to provide longer blood circulation and target-resident time in preclinical studies of fluorescence-guided glioma surgery, such as iron oxide nanoparticles^14–16^, upconversion nanoparticles^17^, and polymer nanoparticles^18–20^.

Silica coating method is a well-known process to provide biocompatible and water-dispersible surfaces to nanoparticles which were synthesized in organic solvent^21–23^. In addition, since the silica shell is optically stable and transparent, it can provide chemically and mechanically stable frameworks for fluorescent dyes by shielding them from external environmental changes^24,25^.

In this research, we developed highly water-dispersible and near-infrared fluorescent silica coated iron oxide nanoparticles (NF-SIONs) as MR/optical combined nanoprobes for *in vivo* cancer imaging. Through successive two-step silica coating process, the hydrophobic iron oxide nanoparticles were converted into nanoparticles with high water dispersibility, strong near-infrared fluorescence properties, and suitable physicochemical properties for biomedical applications. After evaluation of safety and ingestion pattern at the cellular level *in vitro*, we conducted *in vivo* biodistribution study of intravenously administered nanoparticles to a mouse model transplanted with tumor cells in the shoulder or brain via fluorescence imaging. In addition, we also examined the internalization pattern of nanoparticles at tissue level by immunofluorescence analysis using CD31, F4/80, and CD11b, well-known markers that overexpressed at the tumor-associated macrophages. These results demonstrate that the fluorescent silica coated nanoparticles can specifically target tumor-associated macrophages in the microenvironment surrounding primary tumors and they can be used as efficient nanoprobes for fluorescence imaging-guided surgery to improve glioblastoma outcome.

## Results

### Synthesis and characterization of NIR-fluorescent silica coated iron oxide nanoparticles (NF-SIONs)

**Figure 1:**
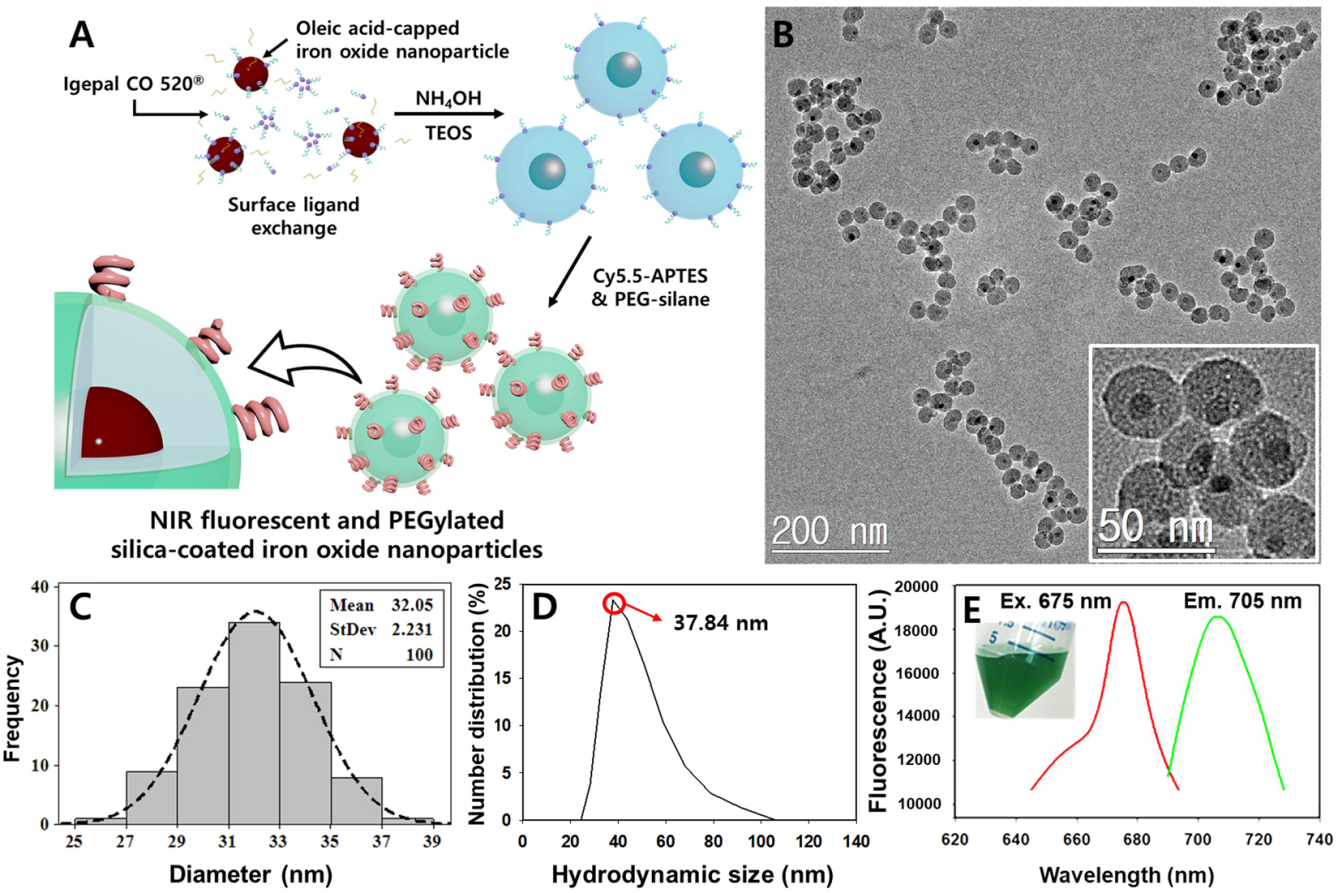
(**A**) Synthetic illustration of NIR-fluorescent silica coated iron oxide nanoparticles (NF-SIONs). (**B**) TEM image of synthesized NF-SIONs. (**C**) Nanoparticle size distribution histogram. (**D**) Hydrodynamic size distribution of NF-SIONs in 0.01M PBS. (**E**) Excitation and emission profile of NF-SIONs.

Near infrared-fluorescent silica shell was coated onto 6 nm-sized iron oxide nanocrystals (Figure S-1) according to the scheme shown in Fig. 1A. First, the ready-made oleic acid-capped monodisperse iron oxide nanoparticles were added to the solvent where the reverse microemulsions exist. Then the Igepal^®^ CO 520, surfactant surrounded the iron oxide nanoparticles, exchanging the surface oleic acid^26^. When the ammonium hydroxide solution was added, the reverse microemulsions expanded and the surfactant coated iron oxide nanoparticles were incorporated into each droplet. After vigorously stirring for 5 minutes, tetraethyl orthosilicate was added and hydrolyzed to form primary silica layer. To achieve the highly sensitive fluorescence imaging through the *in vivo* imaging and immunofluorescence assay, Cy 5.5-labeled aminopropylsilane, and commercial PEG-silane were added to the reaction solution to import NIR fluorescence and dispersion stability. Molecular fluorophores often lose their fluorescence due to photon-induced chemical damages by external light sources, even during the microscopic studies. Thus, structural modification or encapsulation process are commonly used to enhance their fluorescence stability^27^. As shown in Fig. 1B, the overall morphology of synthesized NF-SIONs was observed through TEM analysis and their core/shell structure was clearly observed in magnified image (inset). Their physical size was measured as 32.05 ± 2.23 nm by calculating the average diameter of 100 nanoparticles from transmission electron microscopy images (TEM) and mean hydrodynamic size in number distribution was about 37.84 nm by dynamic light scattering (DLS), meaning that the nanoparticles are well-distributed in aqueous phase (Fig. 1C and D). As can be seen from the fluorescent data of NF-SIONs (Fig. 1E), their excitation and emission spectra were close to that of pristine dye molecules (Flamma^®^ 675 NHS ester, Ex. λ: 675 nm and Em. λ: 691 nm). Since the core iron oxide nanoparticles exhibit strong light absorption in visible wavelength area, it is highly important to control the distance of dye molecules from the iron oxide surfaces to reduce the excitation energy loss^28^. Thus, we primarily coated the iron oxide nanoparticles with pure silica as a physical barrier. These core-shell nanoparticles were further coated with Cy 5.5 and PEG labeled silanes on their outer shell to form a second shell layer.

### Dispersion and fluorescence stability of NF-SIONs

**Figure 2:**
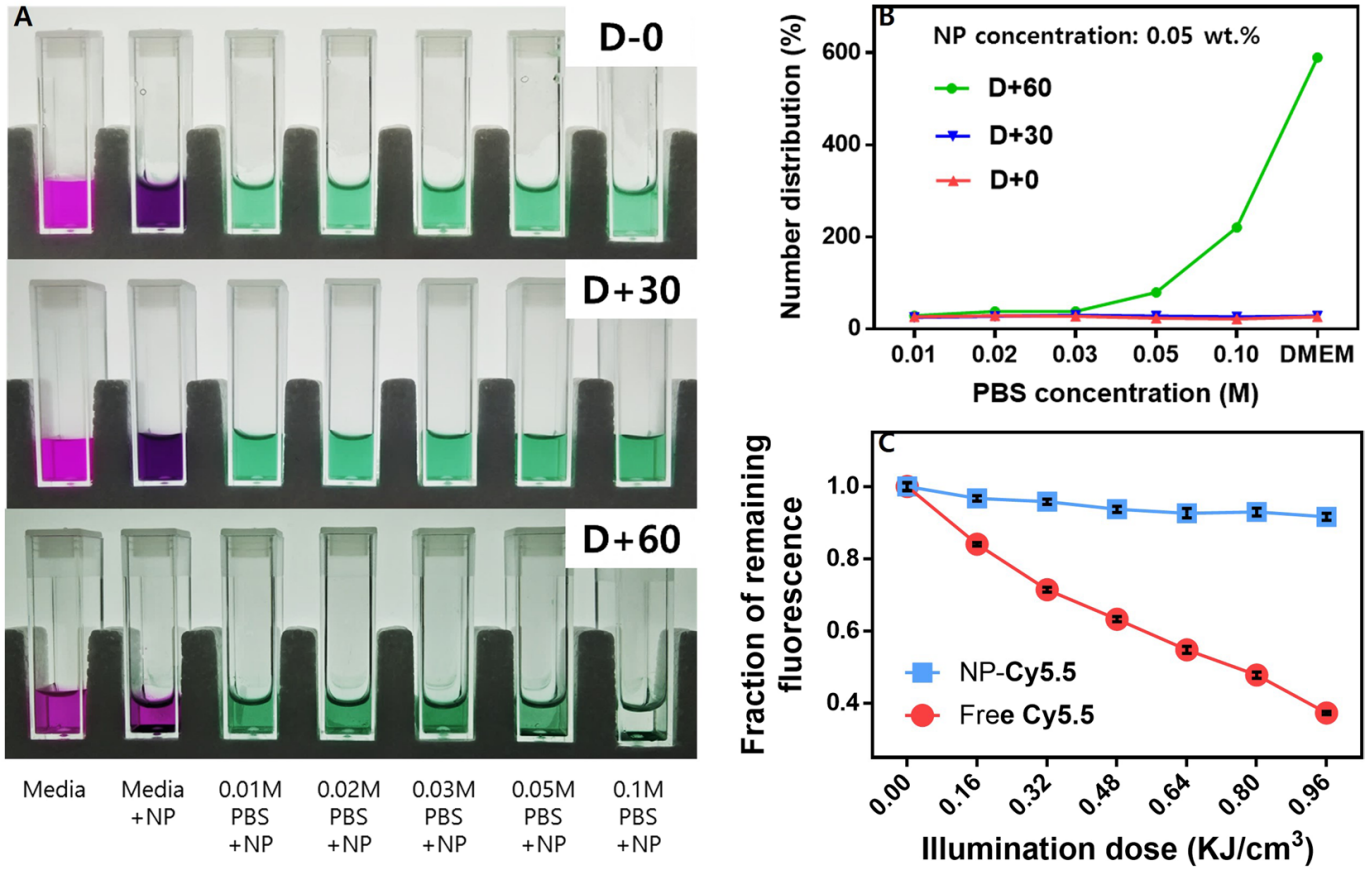
(**A**) Dispersion stability test (~60 days) among cell culture media and PBS with various concentration. (**B**) Hydrodynamic size change observation through DLS measurement. (**C**) Photobleaching comparison of Cy 5.5 dye and NF-SIONs in DI water under xenon light irradiation for 6 minutes. Concentration of nanoparticles was 0.05 wt.% for each.

One of the main obstacles of nanoparticles for bioimaging research is colloidal stability. Since the dispersion of nanoparticles is due to the surface charge in most cases, they are easily aggregated when introduced into buffer solution or body fluid-like media by the non-specific protein adsorption or charge neutralization by counterions. Hence, non-ionic PEG chains are usually used to prevent such irreversible aggregation and enhance the colloidal stability through the circulatory system^29^. In this synthesis, we introduced the PEG chains onto the surface of NF-SIONs after the formation of primary silica layer. To confirm their shelf life among the various conditions, PBS solution and DMEM media were equipped and set up as represented in Fig. 2A. In each disposable cuvette, fresh DMEM media and concentrated PBS solutions (0.01 M to 0.1 M) containing nanoparticles (0.05 wt.%) were transferred and kept in dark, stable area for 2 months. The dispersion state was studied by DLS and the obtained results were summarized in Fig. 2B. For the first 30 days, there was no significant change either from the camera shot or from the DLS number distribution. After 60 days, however, the nanoparticles showed severe aggregation at the 0.1 M PBS and DMEM media. We also compared the fluorescence stability of free-standing dye molecules and dye-incorporated nanoparticles. Each diluted solution was irradiated for a minute with xenon light source and the fluorescence intensity was measured. The experiment was repeated for 6 times and the obtained fluorescence intensity as a function of exposure time was plotted as Fig. 2C after normalization. Using the measured light energy density, we calculated the illumination doses depend on the irradiation time in X-axis. Apparently, we observed the severe fluorescence decay (over 60%) from the free-standing Cy 5.5, while there was no remarkable change (less than 10%) from the colloidal NF-SIONs, meaning that NF-SIONs have superior fluorescence stability compared to free-standing NIR dye. Using the standard fluorescence curve of Cy5.5, the amount of fluorescent dye contained in 1 mg of NF-SIONs was found to be about 12.7 μmol (Figure S-2).

### The magnetization of NF-SIONs and MR phantom imaging

**Figure 3:**
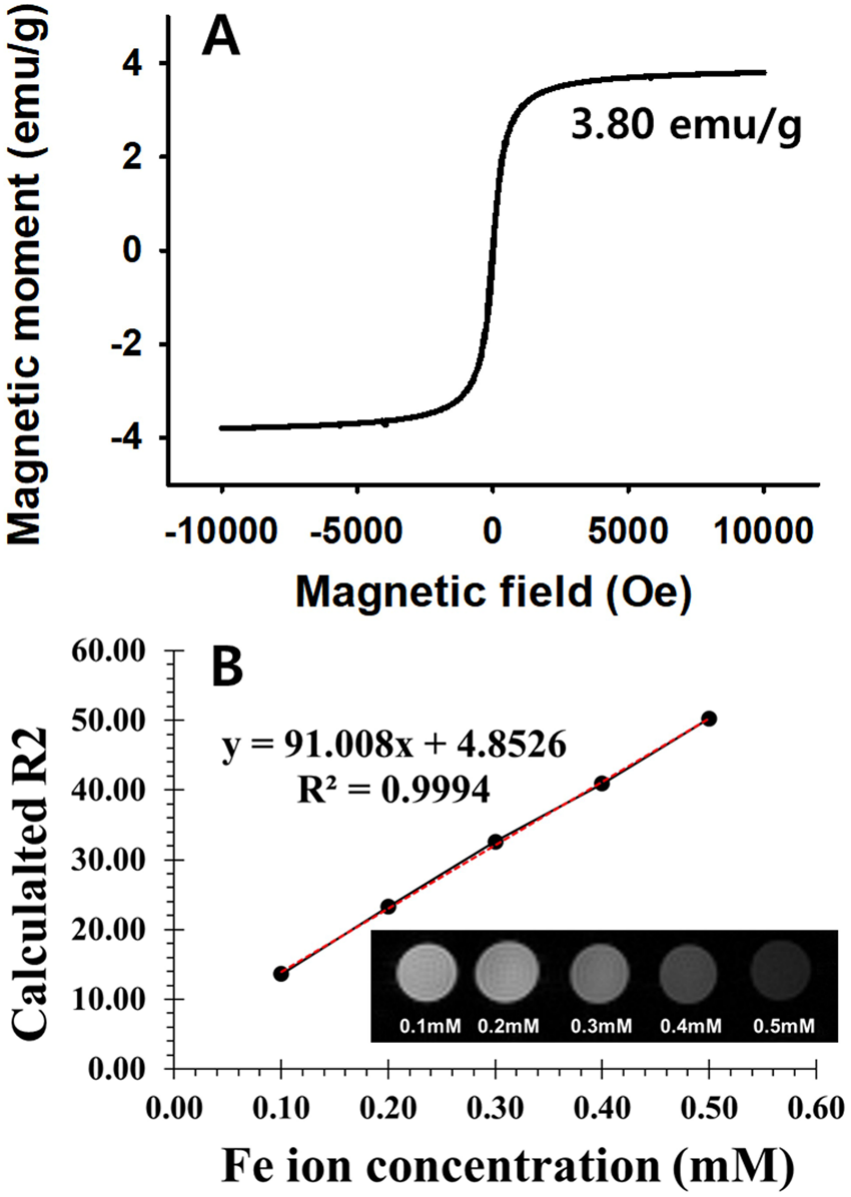
(**A**) SQUID magnetization measurement of the NF-SIONs powder. (**B**) R2 relaxation rates as a function of iron concentration (mM) of NF-SIONs dispersed in DI water, measured at 25 °C and 9.4 T. Inset image represents T2-weighted MR enhancement of NF-SIONs in various concentration.

The magnetic property of NF-SIONs was analyzed by drawing the magnetic hysteresis (M-H) curve in an applied field ranging from −10 ~ 10 kOe at 293 K (Fig. 3A). The M-H curve showed no remnant magnetization after the applied magnetic field was removed, which means that the NF-SIONs are superparamagnetic, and saturation magnetization (Ms) was calculated as 3.80 emu·g^−1^. Such low Ms value is attributed to the diamagnetic contribution of the silica shell, which consists large portion of core-shell nanoparticles^30^. To investigate the MR imaging performance of NF-SIONs, phantom test was conducted following the previously reported method^31^. As plotted in Fig. 3B, serially diluted NF-SIONs showed linear regression in R2 relaxation, and the r_2_ (specific relaxivity) was calculated as 50.36 mM^−1^s^−1^, which is similar or a little less than that of commercialized iron oxide-based contrast agents^32^. These results suggest that our NF-SIONs might be properly used in MR imaging applications.

### Cytotoxicity test of NF-SIONs

**Figure 4:**
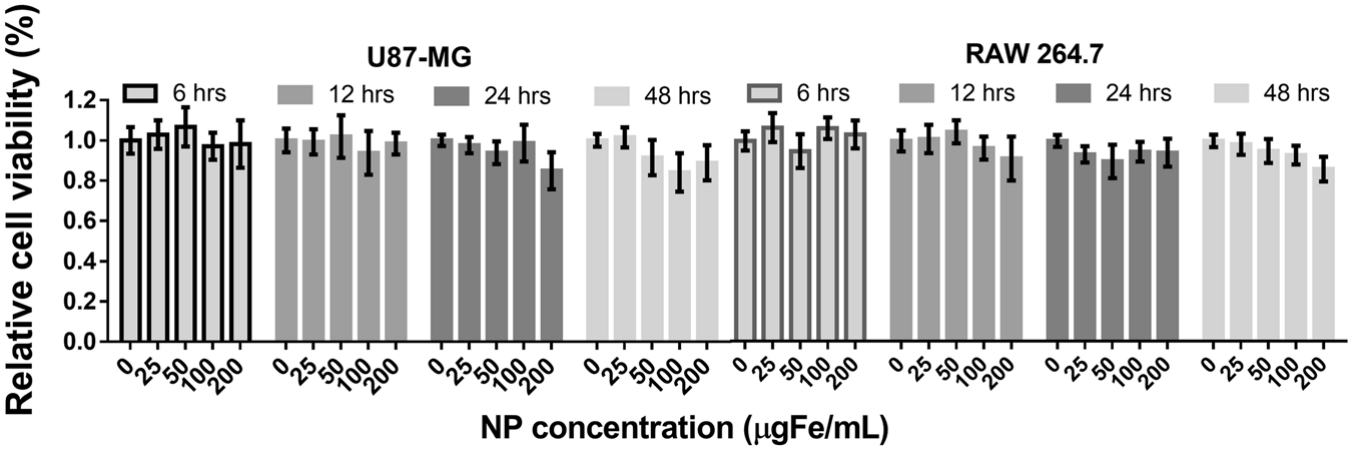
MTT assay results of NF-SIONs against U87-MG and RAW 264.7 cell lines.

The cytotoxicity of silica particles and silica coated iron oxide nanoparticles have been extensively studied in former researches^33,34^. Here, we assessed the cytotoxicity of the as-prepared NF-SIONs from two cell lines (U87-MG; human brain tumor, RAW 264.7; mouse macrophage) prior to *in vivo* bioimaging. As shown in Fig. 4, the viability against the nanoparticles was tested via MTT assay. Overall, the relative viabilities from the two cell lines used in the experiment were inversely proportional to the concentration of nanoparticles and the treatment time. However, more than 90% of the cells survived even at a high concentration (200 μgFe·mL^−1^) and a long incubation time (48 hours), proving that the as-prepared NF-SIONs are biologically safe for further experiments.

### NF-SIONs preferentially stains glioblastoma cells and TAMs *in vitro*

**Figure 5:**
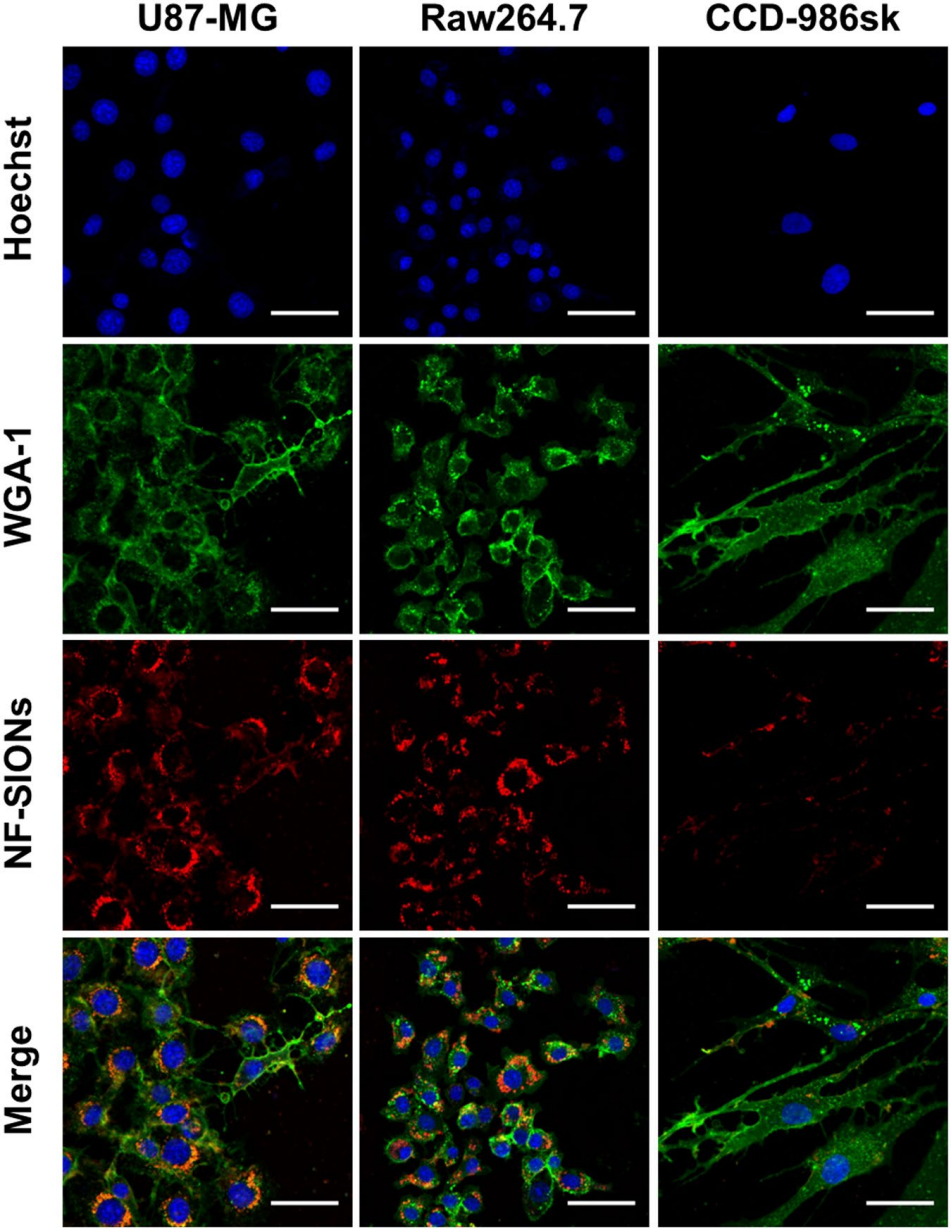
*In vitro* cellular uptake study of U87-MG, RAW 264.7 and CCD-986sk cell lines. Shown are confocal micrographs of cell lines that cultured for 4 hours with nanoparticles-contained medium (10 μgFe·mL^−1^). Scale bars: 50 μm.

Having shown that the adverse effects of the NF-SIONs on cell viability were minimal, we next studied the preferential cellular uptake of NF-SIONs using different cell lines. We were particularly interested in evaluating selective staining ability of NF-SIONs for glioblastoma cells (U87-MG) and tumor-associated macrophages (TAMs, RAW 264.7) as therapeutic targets compared to normal parenchyma cells (CCD-986sk). Confocal microscopy revealed an apparent preference of NF-SIONs for U87-MG glioblastoma cells and RAW 264.7 macrophages over CCD-986sk fibroblasts after 4-hour incubation with 10 μgFe·mL^−1^ of NF-SIONs as shown in Fig. 5. NF-SIONs proved to be robust for probing glioblastoma cells and TAMs selectively *in vitro*, which indicates their *in vivo* targeting potential to delineate glioblastoma.

### NF-SIONs show high uptake for glioblastoma and rapid background clearance through urination in subcutaneous xenograft model by *in vivo* fluorescence imaging

**Figure 6:**
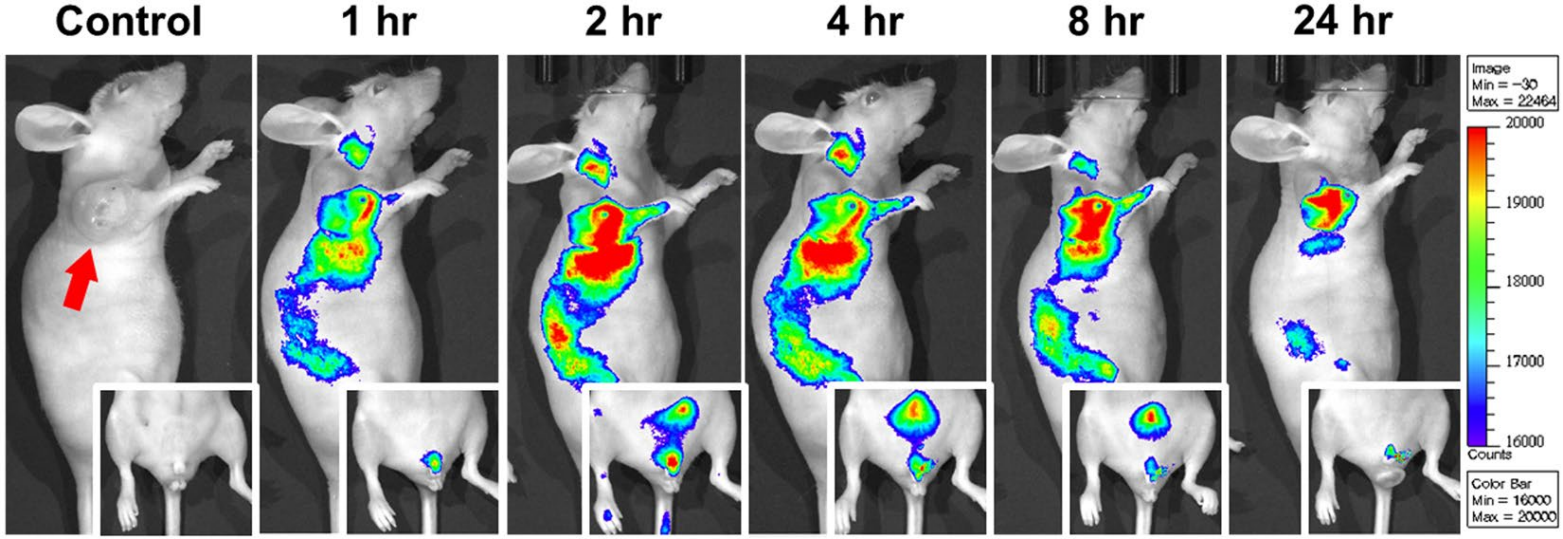
Non-invasive fluorescence imaging of NF-SIONs in the glioblastoma xenograft model for 24 hours to study biodistribution of administered nanoparticles. The inset images show the traces of nanoparticles excreted through the external genitalia.

It is well-known that subcutaneously injected nanoparticles accumulate passively near tumor regions by enhanced permeability and retention (EPR) effects, due to the architectural abnormality of neovascularization and low association with the adjacent lymphatic system^35,36^. Since extremely small nanoparticles (<~10 nm) can be rapidly removed (excreted) by urination and larger particles (>~200 nm) can be removed by the mononuclear phagocyte system (MPS), nanoparticles with intermediate size (10~200 nm) with neutral surface charge and biocompatible coating are highly preferred as suitable bioimaging nanoprobes for efficient vascular delivery^37,38^. Therefore, in this study, NF-SIONs were designed to be in the range of 30–50 nm with a non-ionic surface so that the nanoparticles can not only be transmitted to the whole body including the tumor but also safely excreted via urinary system. As shown in Fig. 6, we injected the synthesized NF-SIONs (200 μgFe) into the tail vein of glioblastoma-bearing mice and performed systemic fluorescence imaging for up to 24 hours. Although it showed significant uptake of nanoparticles not only by tumor but also by liver and intestine up to 8 hour, 24 hour-post image indicated that our nanoprobe successfully delineated glioblastoma tumor (the right shoulder region) with high specificity after thorough clearance from the blood and other organs. Also, as seen in the inset images, the fluorescent signals in the bladder and external genitalia indicated that non-targeted NF-SIONs were well-excreted in the urine.

**Figure 7:**
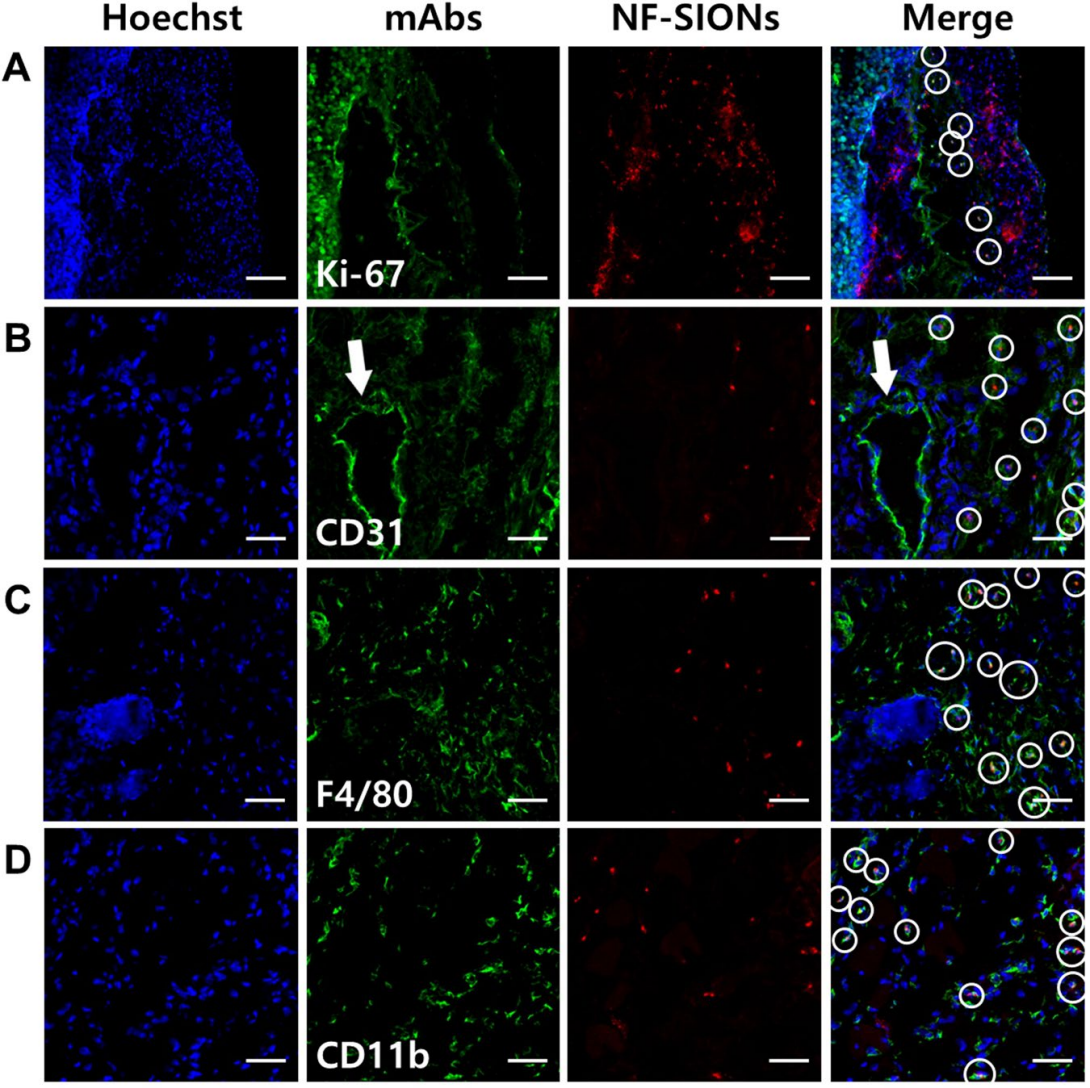
Characterization of targeting and distribution of NF-SIONs in the shoulder tumor region by immunofluorescence staining, 24 hr after injection. The shown sections were stained with monoclonal antibodies (mAbs; green) against Ki-67 (**A**, proliferating cells), CD31 (**B**, endothelial cells), F4/80 (**C**, murine macrophages), and CD11b (**D**, monocytes/macrophages). Blue and red signals show the location of cell nucleus and NF-SIONs, each. White circles indicate co-localization of TAMs and NF-SIONs. Arrow; lumen of blood vessel. Scale bar: 100 μm (×20) for A and 50 μm (×40) for B, C and D.

To analyze the observed fluorescence signals around the tumor with cellular resolution, we excised the tumor and adjacent tissue (24 hours after nanoparticle injection) and performed immunofluorescence study using confocal microscopy, as shown in Fig. 7. Based on the results in Fig. 5, we analyzed the tumoral distribution of nanoparticles using several cellular markers such as Ki-67 (proliferating tumor cells), CD31 (endothelial cells/macrophages), F4/80 (tumor-associated macrophages), and CD11b (monocytes/macrophages) as shown in Fig. 7. Interestingly, most of the nanoparticles-binding cells expressed macrophage related F4/80 and CD11b. The nanoparticle signal, however, did not colocalize with Ki-67 positive tumor cells and CD31 positive endothelial cells surrounding the lumen of blood vessel (an arrow). This is contrary to the fact that the cellular uptake study is shown in Fig. 5, which did not reveal a large difference in nanoparticle internalization between tumor cells and macrophage cell lines. This may have induced due to differential uptake ability of general macrophages and tumor-associated macrophages which were activated by secretion of chemokines from tumor. Although cellular targeting ability of the developed nanoprobe did not show prominent difference between tumor cells and macrophages under *in vitro* condition, their *in vivo* specificity for activated tumor-associated macrophages over glioblastoma cells was successfully confirmed by immunohistological analysis. Therefore, we concluded that the nanoparticles injected into the body were well accumulated around the tumor due to the EPR effect through neovasculature, but they were mostly taken up by tumor-associated macrophages, activated and endocytosis enhanced macrophages due to several chemokines secreted from tumor, rather than by the tumor cells.

### NF-SIONs penetrate the blood-brain barrier and delineate glioblastoma specifically in orthotopic xenograft model by *in vivo* fluorescence imaging

**Figure 8:**
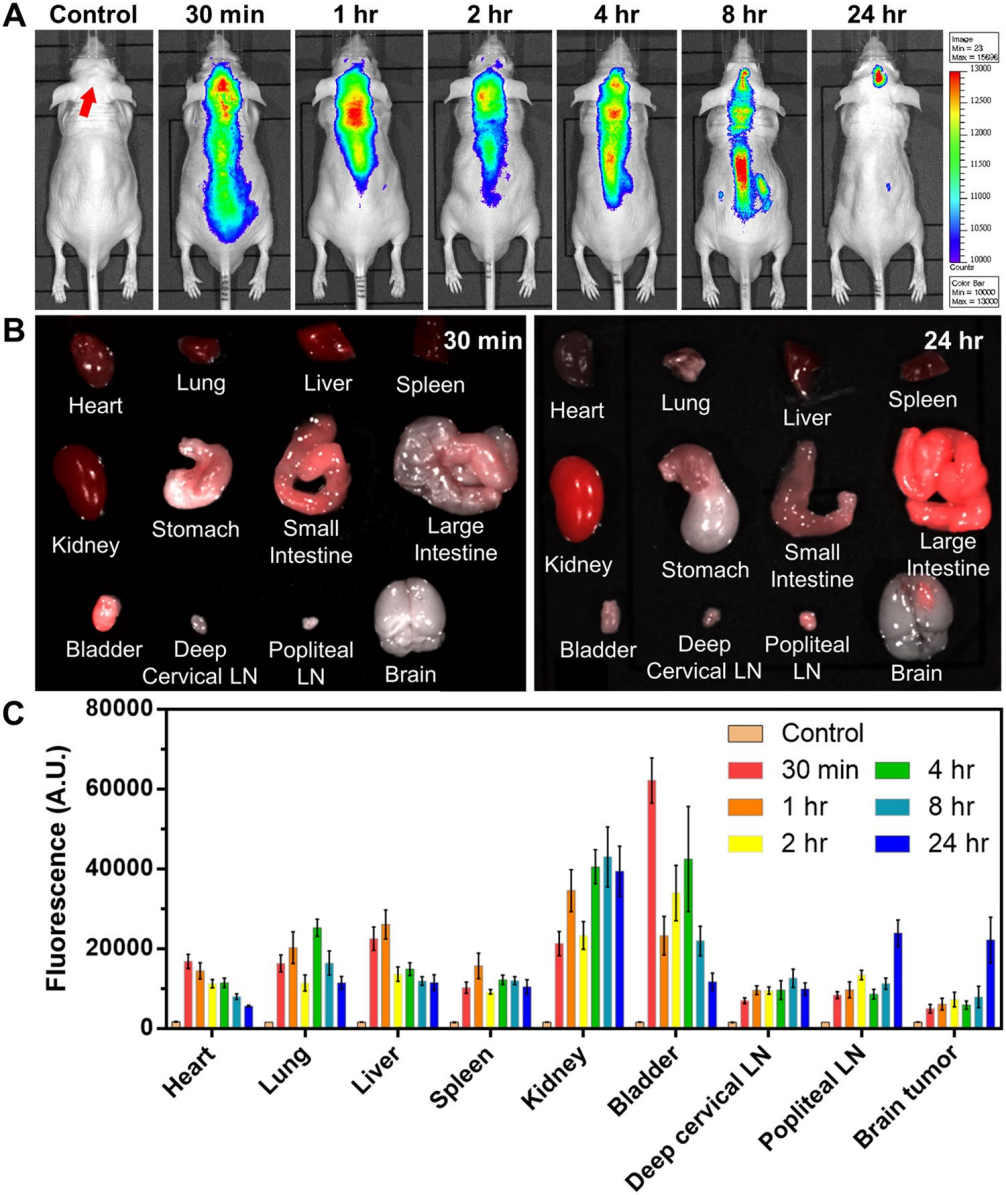
(**A**) Biodistribution study of NF-SIONs in the orthotopic model among 24 hours by non-invasive fluorescence imaging. (**B**) Analysis of fluorescence intensities at 30 mins and 24 hours after injection. (**C**) Target-to-background ratio (TBR) comparison of fluorescence intensity for each organ by the time taken after injection (FOV: 7.5).

Considering the primary nature of the U87-MG cell line, in-depth comparisons were performed to confirm the specific uptake of nanoparticles in the orthotopic model as well as in the subcutaneous model. Unlike elsewhere, the central nervous system is protected by a robust defense system called blood-brain barrier (BBB), which is a major obstacle to the development of drugs and nanoparticles for brain-related diseases^39^. However, recent studies of the relationship between glioma and BBB have shown that tumor cells can disrupt the BBB system and damage the tight junctions, allowing hydrophilic nanoparticles to enter^40–43^. Therefore, it was expected that NF-SIONs with high dispersion stability and hydrophilicity will flow into the brain through the damaged BBB despite its relatively large size. As in the imaging study with subcutaneous xenograft models, NF-SIONs were injected through the tail vein, and the fluorescence signals in the whole body and each organ were analyzed up to 24 hours at regular intervals, as shown in Fig. 8. Intracranial glioblastoma uptake by NF-SIONs showed the peak signal 24 hours after injection as depicted in Fig. 8A,B. Non-targeted NF-SIONs were smoothly excreted through the kidneys and the bladder (urine) confirmed by *ex vivo* fluorescence signal analysis (TBR data in Fig. 8C). Although abdominal organ uptake is still high at 24 hour-post injection, 24 hour-post injection image showed obvious contrast between glioblastoma region and normal brain parenchyma, providing a decent tool for intraoperative guidance of glioblastoma resection.

**Figure 9:**
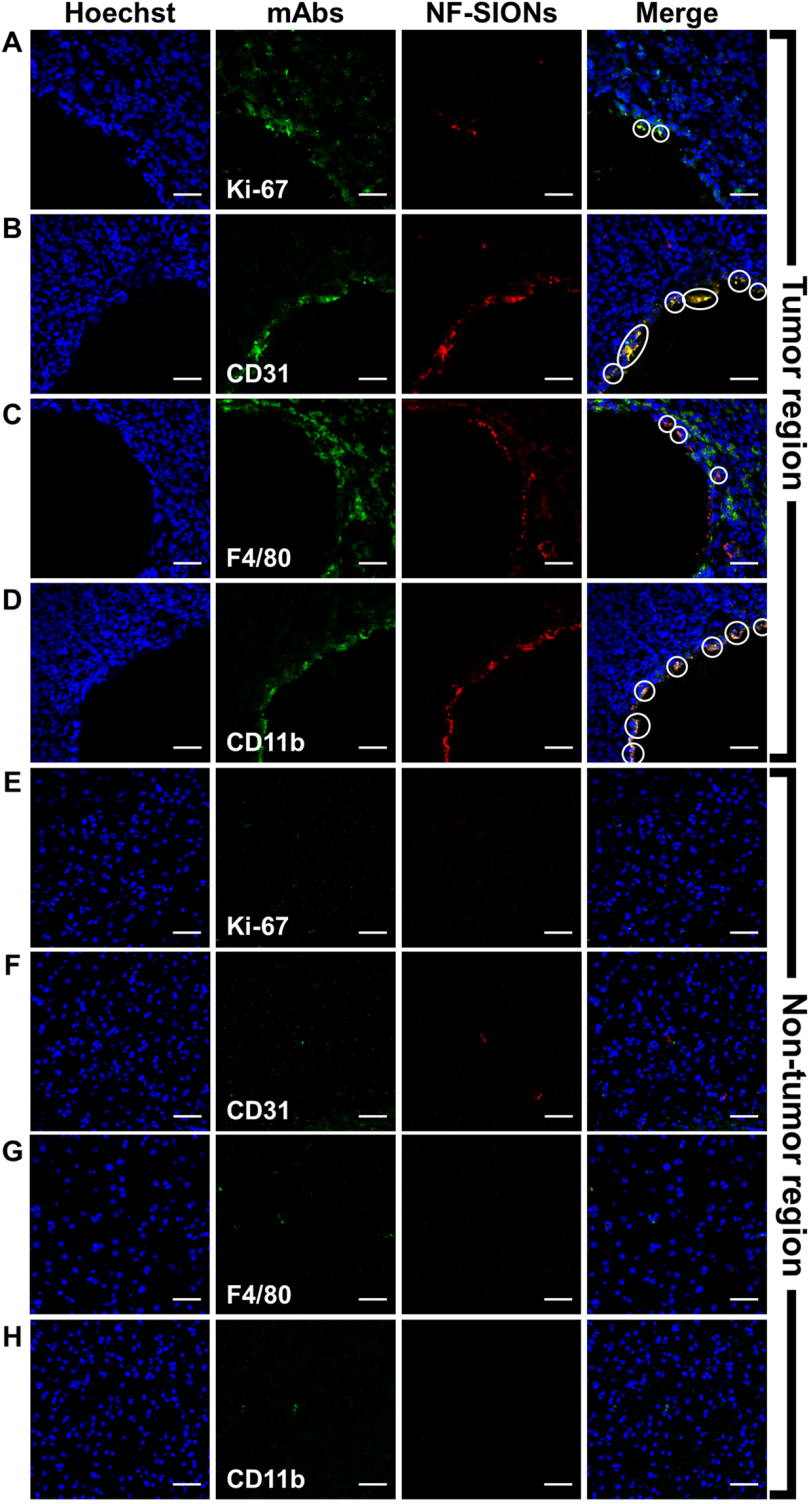
Characterization of targeting and distribution of NF-SIONs in the brain tumor region (**A**–**D**) and non-tumor region (**E**–**H**) by immunofluorescence staining, 8 hr after injection. The shown sections were stained with monoclonal antibodies (mAbs; green) against Ki-67 (**A** and **E**, proliferating cells), CD31 (**B** and **F**, endothelial cells/macrophages), F4/80 (**C** and **G**, macrophages), and CD11b (**D** and **H**, monocytes/macrophages). Blue and red signals show the location of cell nucleus and NF-SIONs, each. White circles indicate co-localization of TAMs and NF-SIONs, and the hole seen in the tumor region was due to the injection process of tumor cells. Scale bar: 50 μm (×40).

**Figure 10:**
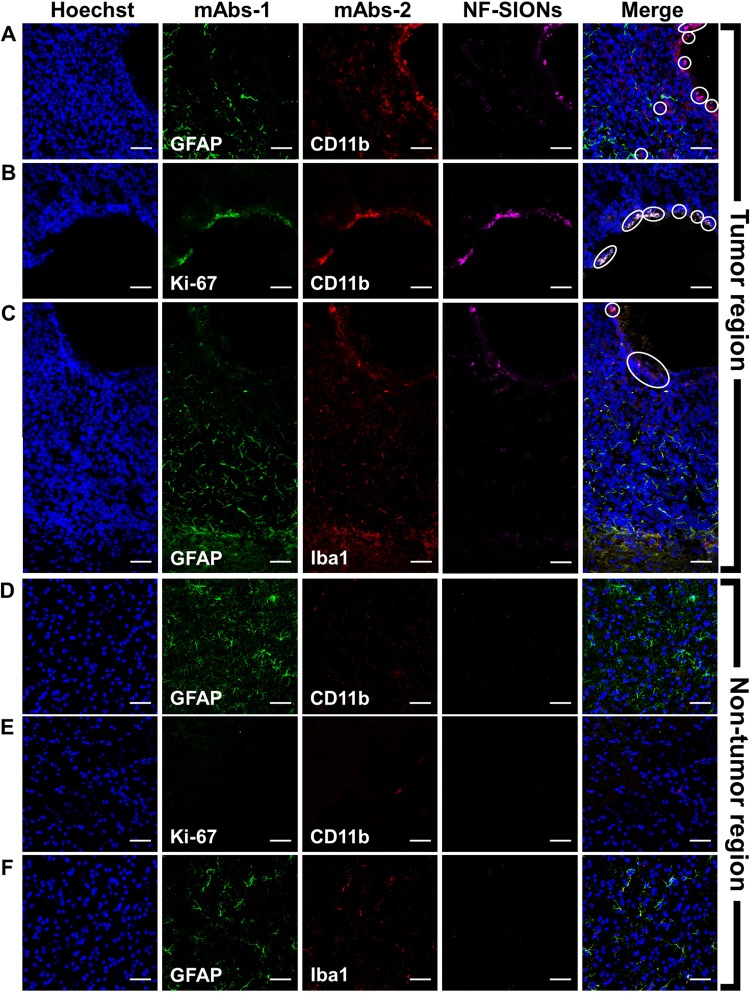
Characterization of targeting and distribution of NF-SIONs in the brain tumor region (**A**–**C**) and non-tumor region (E 9G) by immunofluorescence staining, 8 hr after injection. The shown sections were stained each with two monoclonal antibodies (mAbs; green and red) against GFAP (**A**,**C**,**D** and **F**, astrocyte), CD11b (**A**,**B**,**D** and **E**, monocytes/macrophages), and Iba1(C and F, microglia). Compared with the non-tumoral region, macrophages and microglial cells were highly expressed in the tumor region, and nanoparticles-binding cells were well overlapped with tumor-associated macrophages/microglias (CD11b^+^ or Iba-1^+^ cells), but not with astrocytes (GFAP^+^ cells). Blue and magenta signals show the location of cell nucleus and NF-SIONs, each. White circles indicate co-localization of TAMs and NF-SIONs, and the hole seen in the tumor region was due to the injection process of tumor cells. Scale bar: 50 μm (×40).

Having shown the ability to specifically detect glioblastoma with NF-SIONs via *in vivo* whole body and *ex vivo* organ imaging, we proceeded to identify the characteristics of the NF-SIONs’ cellular localization by immunohistofluorescence staining of excised brain in the orthotopic model, 8 hours after injection (Figs 9 and 10). The *ex vivo* fluorescence signal was observed to be strongest in the brain 24 hours after injection, but at the tissue level fluorescence analysis, the brain sample at 8 hours after injection showed better results. Therefore, the imunofluorescence staining results from excised brain of 24 hour post-injection were summarized in Figure S-4 and S-5. We compared distribution of NF-SIONs in the right and the left sides of brain corresponding to the tumor and the non-tumor regions, respectively, to verify *in vivo* and *ex vivo* imaging data. Indeed, most NF-SIONs binding cells were localized in the tumor region (Figs 9A and 10A) showing specific targeting of glioblastoma area whereas almost no NF-SION positive cells were found in normal brain region. Specifically, most of the NF-SIONs bound to macrophages (CD31^+^ or F4/80^+^ or CD11b^+^) or microglias (Iba1^+^, brain macrophages), but not astrocytes (GFAP) as shown in Figs 9 and 10. Some of the Ki-67^+^ cells, indicative of proliferating cancer cells, were overlapped with the NF-SION^+^ cells as presented in Fig. 9A, however, further examination is required because they might be proliferating inflammatory cells (Ki-67^+^ and CD11b^+^ cells) as confirmed by dual staining with CD11b monocytes/macrophages marker in Fig. 10B. Overall immunofluorescence analysis showed that the injected NF-SIONs were selectively caught by tumoral region compared to non-tumor region, and they were specifically taken up by the tumor-associated immune cells (monocytes/macrophages/microglias) over brain parenchyma cells (astrocytes).

## Discussion

In this study, we introduced a facile synthesis of dual-modal imaging nanoparticles with improved dispersibility and robust fluorescence properties and demonstrated their application as tumor-associated macrophage-specific probes for fluorescence guided surgery of glioblastoma in murine xenograft models. After confirmation the suitability of the NF-SIONs for *in vivo* experiments via MTT assay and cell uptake test, *in vivo* fluorescence imaging was performed in subcutaneous and orthotopic xenograft models to analyze time-course *in vivo* behaviors of nanoparticles and their uptake pattern by the immune cells in the tumor tissues. By performing *in vivo* fluorescence analysis for 24 hours, we demonstrated that the administered nanoparticles were well excreted in the urine and remained only in the tumoral region. In addition, immunofluorescence staining using various monoclonal antibodies showed that the injected nanoparticles exhibited a high uptake in tumor-associated immune cells (monocytes/macrophages/microglias) over cancer cells and brain parenchymal cells. Overall we provide the NF-SIONs as a valuable tool to improve surgical outcomes for glioblastoma patients by providing accurate delineation of tumor margins via specific targeting of tumor-associated macrophages during glioblastoma surgery.

Glioblastoma is a deadly cancer due to their invasive and infiltrative features. For complete surgical resection of glioblastoma which is the most effective therapeutic option, we developed an *in vivo* fluorescence imaging technique using highly water dispersible and fluorescently stable NF-SIONs and successfully demonstrated their feasibility to guide tumor margins to surgeons. Although nanomaterial-based approach to imaging macrophages given their naturally high endocytosis activity is well-known, there have been no trial to use this technique to surgically visualize glioblastoma margins focusing on specific localization of tumor-associated macrophage in the tumor boundary and their significant role for tumor prognosis. Clinical trials of molecular imaging technique using nanoparticles is now vibrant such as C dots based optical-PET imaging^44^. Future study to show the potential of our developed nanoprobe for MRI based glioblastoma diagnosis will booster clinical translation of tumor-associated macrophage targeting NF-SION imaging to provide a one-shot serial imaging strategy from preoperative diagnosis to intraoperative guidance. This will make the clinical management of glioblastoma more effective.

## Methods

### Synthesis of NIR-fluorescent silica coated iron oxide nanoparticles (NF-SIONs)

A typical synthesis of silica coating process on iron oxide nanoparticles was referred to previous papers^23,26,45^. In 100 mL glass vial, cyclohexane (45 mL) and IGEPAL^®^ CO-520 (2.3 g) were added and magnetically stirred for 5 minutes. Then, the prepared colloidal iron oxide solution (500 μL) and ammonium hydroxide solution (600 μL) were serially dropped into the solution by 5 minutes’ intervals. It became blurred instantly but recovered to the transparent solution soon. After the addition of TEOS (150 μL), it was kept for 10 hours with mild stirring. *In situ* PEGylation and NIR dye-labeling was achieved by injection of ready-made NIR dye-APTES complex and commercial PEG-silane solution. In brief, flamma 675-NHS ester (10 μmol) and APTES (200 μmol) were mixed in methanol (1 mL) to form Cy5.5-APTES complex and stirred during 24 hours in the fridge. After 10-hour silication process, the Cy5.5-APTES complex solution (100 μL) and SIH 6188.0 (400 μL) was serially added to the reaction mixture under mild stirring for 2 hours. The reaction was suddenly interrupted by acetone (30 mL) and soon, the aggregation of silica coated nanoparticles was observed, and they were easily collected via centrifugation. The nanoparticles were fully redispersed in ethanol and precipitated with diethyl ether. Repeated twice, the collected nanoparticles were dialyzed in 0.01 M PBS solution for overnight to remove any residual solvents. Finally, the concentration of nanoparticles was set to 1 mgFe·mL^−1^ for further *in vitro* and *in vivo* experiments.

### Photo-stability tests

Comparative study regarding the photo-stable characteristic of pristine dye and dye-loaded nanoparticles was conducted using xenon arc light source (Lambda XL, Sutter instrument, USA). As shown in Fig. S-3, the distance between the light source and the cuvette was fixed. Then, the fluorescence intensity from two cuvettes with a dye-only solution and colloidal NF-SIONs solution was measured at 675 nm wavelength by 1 minutes’ interval under continuous illumination. The amount of Cy 5.5 in each solution was set to be identical, based on the calculated amount of loaded dye in NF-SIONs. The initial light intensity was measured by an optical power meter, then divided by the illuminated volume at the same position. The calculated power per unit volume was 2.65 W·cm^−3^ and hence the accumulated illumination dose was calculated as below.

$${\rm{Illumination}}\,{\rm{dose}}\,[{\rm{J}}\,\cdot \,{{\rm{cm}}}^{-3}]={\rm{Light}}\,{\rm{power}}\,{\rm{per}}\,{\rm{volume}}\,[{\rm{W}}\,\cdot \,{{\rm{cm}}}^{-3}]\times {\rm{Illumination}}\,{\rm{time}}\,[{\rm{s}}]$$

### Dispersion stability tests

Dispersion stability of as-prepared nanoparticles was tested in cell media and PBS buffer solution with various concentration. The concentration of nanoparticles was set to 20 µgFe·mL^−1^ and each nanoparticle-containing solution was placed in a disposable cuvette. To prevent the evaporation, the cuvettes were capped with parafilm and kept in the dark area. By 30 days, DLS measurement was conducted after gentle shaking of cuvettes and each peak value was sorted in a graph.

### MR phantom study

MR phantom images were taken using a 9.4 T/160 A animal MRI system (Agilent Technologies, Santa Clara, CA, USA) in T2 mapping mode. After the collection of T2 from each concentration, we plotted the linear relationship between the R2 (=1/T2) and [Fe]. The transverse relaxation time was estimated by using MEMS (multi-echo multiple slices) sequences with a spin-echo readout. The detail sequence parameters were as follows: TR = 3000 ms, TE = 8.36 ms, NE = 16, average = 1, matrix = 128 × 128, FOV (Field of View) = 65.0 × 65.0 mm^2^, slice thickness = 2.0 mm and scan time = 6.5 min.

### *In vitro* uptake tests

Cells were seeded in 10 mm-cover glass bottom dishes at a density of 5 × 10^4^, 24 hours before *in vitro* experiments. Nanoparticles were treated with serum containing media to the cells by 10 μgFe·mL^−1^ at 37 °C for 4 hours. For cell nucleus and membrane staining, cells were soaked with phosphate buffered saline (PBS) gently so as not to be detached. Shortly afterward, cells were incubated with Hoechst (H3570, 1:500; Life Technologies, USA) in serum-free medium at 37 °C for 20 minutes and then with Wheat Germ Agglutinin (W11261, 5 μg·mL^−1^; Life Technologies, USA) under the same condition as Hoechst staining for 10 minutes. Cells were visualized by a confocal microscope with x20 magnification.

### *In vivo* fluorescence imaging

All animal experiments were carried out in accordance with the approved guidelines. All animal experimental protocols were approved by the Institutional Animal Care and Use Committee of Preclinical Research Institute in the Seoul National University Bundang Hospital (15099). Animals were anesthetized by 2% isoflurane gas and nanoparticles were administered intravenously with an insulin syringe. *In vivo* fluorescence images were acquired using *In vivo* Imaging System with the indicated wavelength (excitation: 660 nm, emission: 710 nm). Mice were kept alive and maintained body temperature at 37 °C during the imaging experiment. All images were analyzed by ImageJ software in terms of calculation of target-to-background (TBR).

## Electronic supplementary material

Material characterization and additional data: core particle size distribution, fluorescence quantification, optical stability configuration and additional in vivo immunofluorescence images
