# Supplementary material for: In vivo delineation of glioblastoma by targeting tumor-associated macrophages with near-infrared fluorescent silica coated iron oxide nanoparticles in orthotopic xenografts for surgical guidance
Source: Sci Rep. 2018 Jul 24;8:11122. doi: 10.1038/s41598-018-29424-4 (PMC6057886; doi:10.1038/s41598-018-29424-4)
Supplement: Supplementary file 1 — Material characterization and additional data: core particle size distribution, fluorescence quantification, optical stability configuration and additional in vivo immunofluorescence images [file 41598_2018_29424_MOESM1_ESM.docx]

**Supporting Information**

***In vivo* delineation of glioblastoma by targeting tumor-associated macrophages with near-infrared fluorescent silica coated iron oxide nanoparticles in orthotopic xenografts for surgical guidance**

*Chaedong Lee,^1^ Ga Ram Kim,^1,2^ Juhwan Yoon,^3^ Sang Eun Kim,^1,2,3^ Jung Sun Yoo,^*4^ Yuanzhe Piao^*1,3^*

*^1^ Department of Transdisciplinary Studies, Seoul National University, Republic of Korea.*

*^2^ Department of Nuclear Medicine, Seoul National University Bundang Hospital, Republic of Korea.*

*^3^ Advanced Institutes of Convergence Technology, Republic of Korea.*

*^4^ Department of Health Technology and Informatics, The Hong Kong Polytechnic University, Hong Kong SAR, P. R. China.*

Correspondence and requests for materials should be addressed to Y.P. (email: parkat9@snu.ac.kr) or to J.S.Y. (email: jungsun.yoo@polyu.edu.hk).

**Supplementary Figures and Tables**

**Figure S-1.** (A) Transmission electron micrograph of core hydrophobic iron oxide nanoparticles and (B) a histogram plotted according to their physical size distribution.


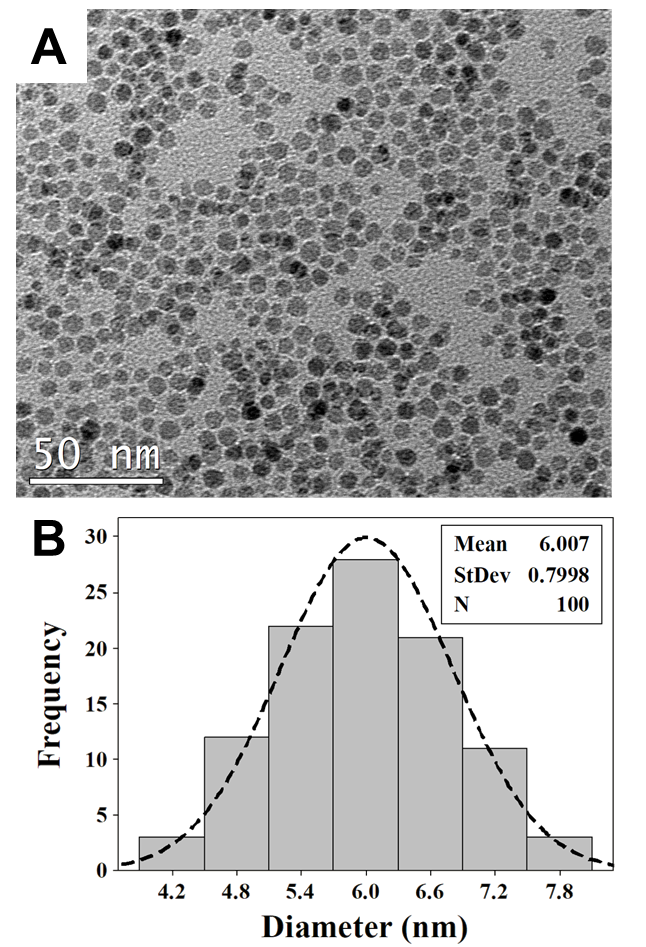

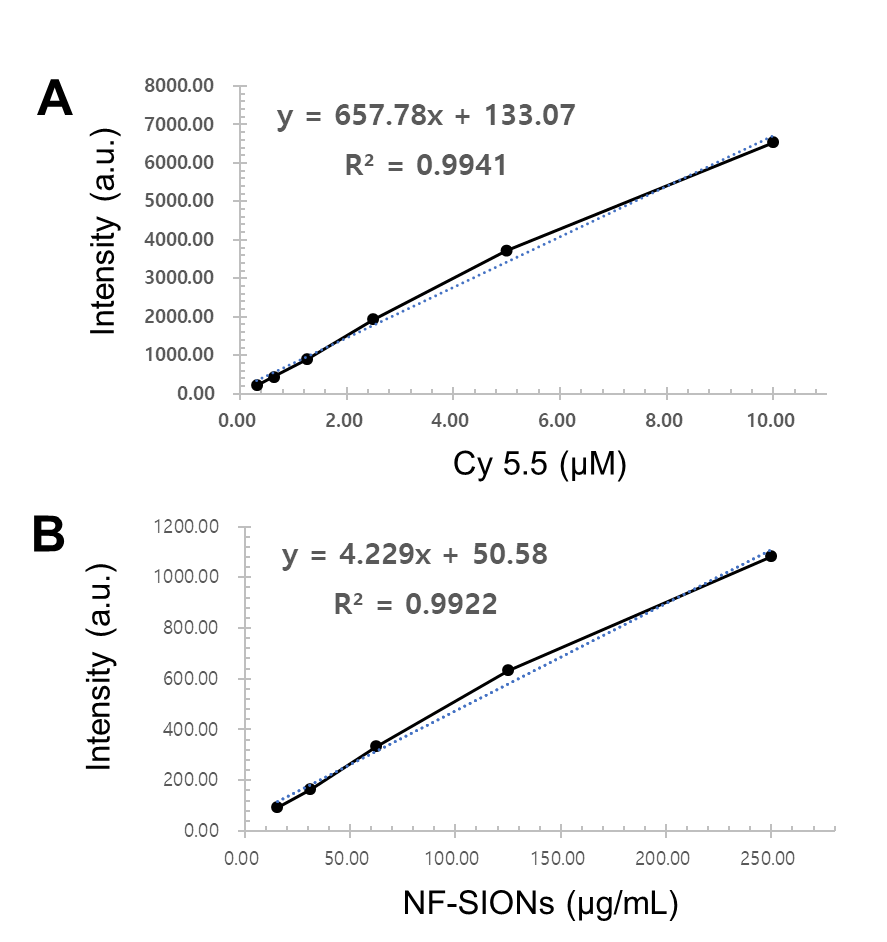

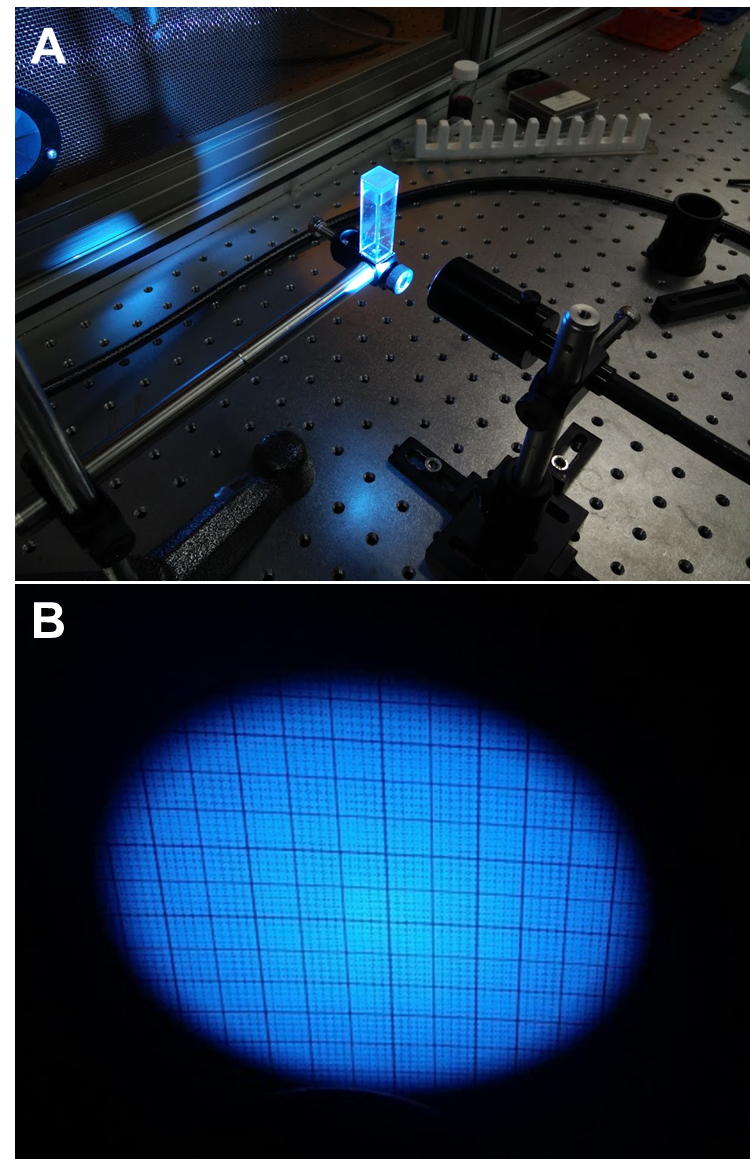

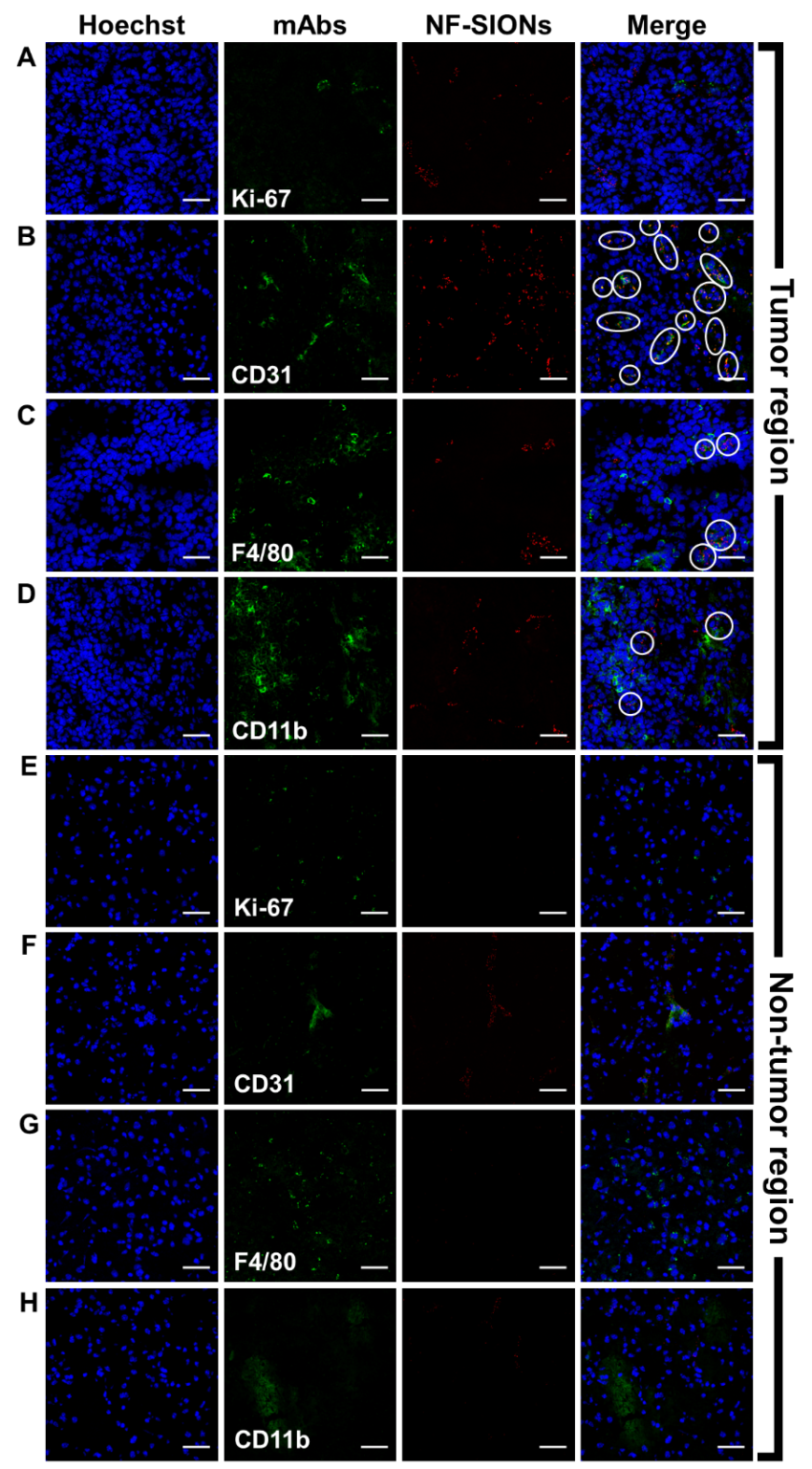

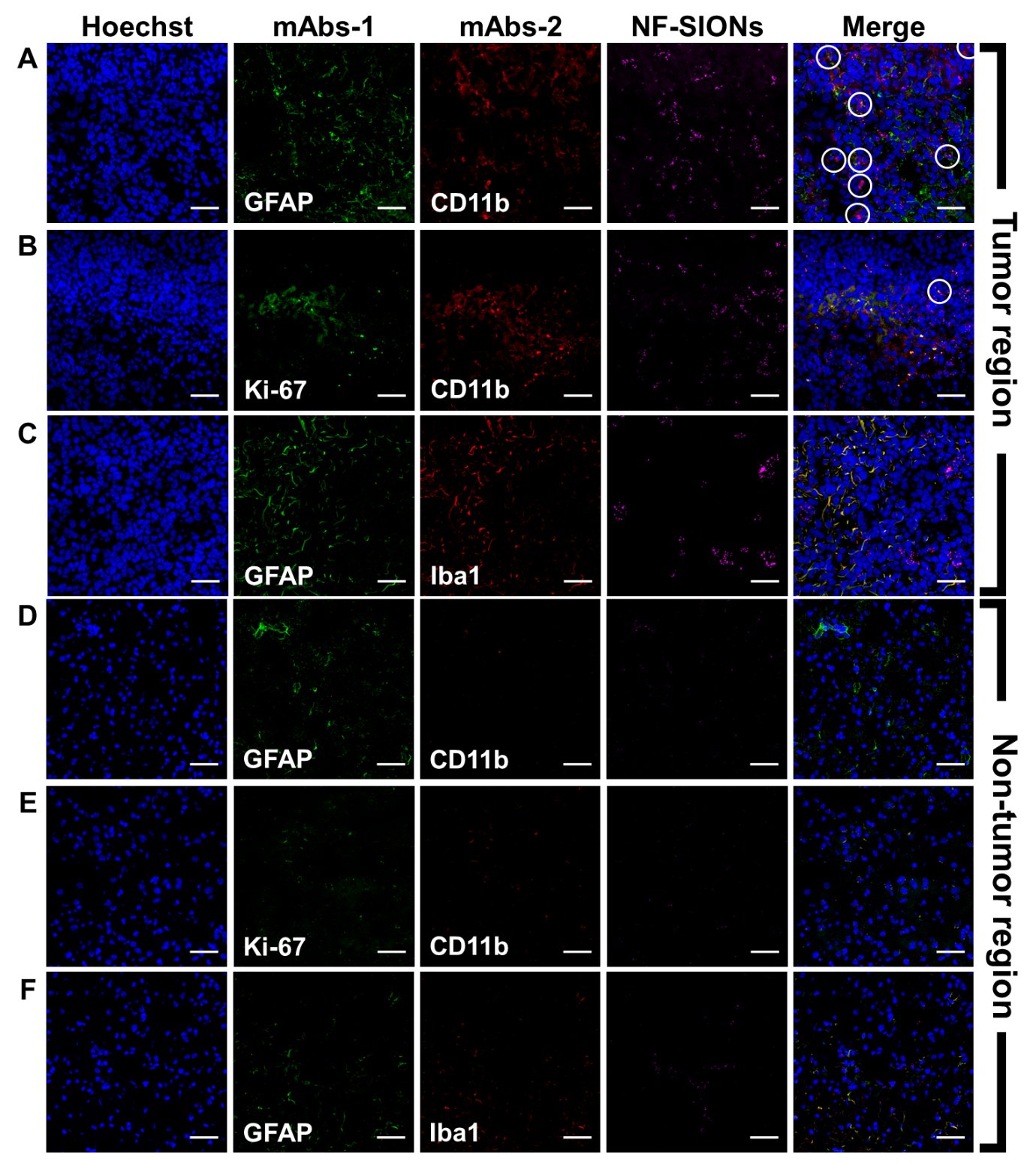


**Figure S-2.** Standard curves of fluorescence intensity from (A) Cy 5.5 stock solution and (B) NF-SIONs. By comparing the fluorescence intensities according to the respective concentrations, it was confirmed that 1 mg of nanoparticles contain about 12.7 μmol of Cy5.5 dye molecules.

**Figure S-3.** (A) Xenon lamp (Lambda XL, Sutter instrument, USA) radiation set up using transparent disposable cuvette. (B) Light power per area was measured using grid paper.

**Figure S-4.** Characterization of targeting and distribution of NF-SIONs in the brain tumor region (**A, B, C** and **D**) and non-tumor region (**E, F, G** and **H**) by immunofluorescence staining, **24 hr after injection**. The shown sections were stained with monoclonal antibodies (mAbs; green) against Ki-67 (**A** and **E**, proliferating cells), CD31 (**B** and **F**, endothelial cells), F4/80 (**C** and **G**, murine macrophages), and CD11b (**D** and **H**, monocytes/macrophages). Despite the strong fluorescence signal observed in the ex-vivo fluorescence image (**Figure 8B.**), it was confirmed through confocal microscopy that the frequency of expressed TAMs and the number of ingested nanoparticles in the tumor region were relatively small. Blue and red signals show the location of cell nucleus and NF-SIONs, each. White circles indicate co-localization of TAMs and NF-SIONs. Scale bar: 50 μm (×40)

**Figure S-5.** Characterization of targeting and distribution of NF-SIONs in the brain tumor region (**A, B** and **C**) and non-tumor region (**E, F** and **G**) by immunofluorescence staining, 24 hr after injection. The shown sections were stained each with two monoclonal antibodies (mAbs; green and red) against GFAP (**A, C, D** and **F**, astrocyte), CD11b (**A, B, D** and **E**, monocytes/macrophages), and Iba1(**C** and **F**, microglia) to compare the nanoparticles uptake among tumor-associated macrophages and unrelated cells simultaneously. Compared with the non-tumoral region, macrophages and microglial cells were highly expressed in the tumor region, and some of nanoparticles were found in tumor-associated macrophages, such as CD11b, rather than GFAP, Ki-67 and Iba-1, which constitute brain tissue. Blue and magenta signals show the location of cell nucleus and NF-SIONs, each. White circles indicate co-localization of TAMs and NF-SIONs. Scale bar: 50 μm (×40)

**Supplementary Methods**

**Preparation of 8 nm-sized iron oxide nanocrystals.** In order to acquire dual-functional imaging nanoprobes with MR and NIR fluorescent bimodality, we obtained iron oxide nanocrystals following previously reported method with minor modifications ^1^. Typically, Fe(acac)_3_ (350 mg), 1, 2-hexadecanediol (1.29 g), oleylamine (0.8 g), oleic acid (0.85 g) and benzyl ether (10 mL) were transferred into three-neck round bottom flask and magnetically stirred for 30 minutes. Residual moisture was removed by low-pressure degassing at 100 ℃ for an hour. Then the mixture was heated to 300 ℃ (reflux) for an hour under Ar gas atmosphere. The resulted black solution was cooled down and acetone was added to precipitate the synthesized nanocrystals. The nanoparticles were collected at 8000 rcf centrifugation and the black sediment was recovered with n-hexane. The same precipitation and centrifugation procedure was repeated twice and finally the nanoparticles were dispersed in cyclohexane (~5 mg·mL^-1^).

**Materials.** 3-(4,5-dimethylthiazol-2-yl)-2,5-diphenyltetrazolium bromide (MTT), 1,2-hexadecanediol, oleylamine (70%), benzyl ether, tetraethyl orthosilicate (TEOS), IGEPAL® CO-520, (3-Aminopropyl)triethoxysilane (APTES), dimethyl sulfoxide and phosphate buffered saline (PBS, tablet) were purchased from Sigma Aldrich. Ammonium hydroxide (28~30 wt.%), ethanol, methanol, acetone, n-hexane, cyclohexane, oleic acid and diethylether was provided by Samchun chemicals (South Korea). SIH 6188.0 ([Hydroxy(polyethyleneoxy)propyl]triethoxysilane, 50% in ethanol) was provided by Gelest and iron(Ⅲ) acetylacetonate was purchased from Strem chemicals. Flamma 675-NHS ester was from Bioacts (South Korea). All chemicals were used without any purification process.

**Characterization.** Transmission electron microscopy (TEM) images were obtained from LIBRA 120 (Carl Zeiss) at an accelerating voltage of 120 kV and the hydrodynamic size distribution measurement was conducted by using DLS (Zetasizer Nano ZS, Malvern instruments) equipped with a He–Ne laser operating at 633 nm and a back-scattering detector at 173°. Fluorescence excitation and emission spectra of NF-SIONs were studied through Photoluminescence spectroscopy (FluoroMate FS-2, Scinco). Inductively coupled plasma-emission spectrometer (ICPS-7500, Shimadzu) was used to quantify the amount of iron of the NF-SIONs and their magnetization curve against the external magnetic field was obtained via PPMS-14 (Quantum design). *In vivo* and ex vivo fluorescence imaging of mouse model was achieved by using IVIS (Lumina XRMS; CLS136340, Perkin Elmer) and *in vitro* cell uptake and IHC assay was studied with confocal microscope (A1 Rsi, Nikon).

**Cell lines.** Human malignant glioblastoma cell U87-MG and murine monocyte RAW 264.7 were distributed from Korean Cell Line Bank (Seoul, Korea) and grown in Dulbecco’s Modified Eagle’s Medium (DMEM, SH30243.01; GE Healthcare, USA) supplemented with 10% heat-activated fetal bovine serum (FBS, SH30919.03; GE Healthcare, USA) and 1% penicillin-streptomycin (15070-063; Life Technologies, USA). U87-MG cell line stably expressing firefly luciferase named U87-MG-luc was established as previously described for *in vivo* bioluminescence imaging of glioblastoma orthotopic models ^2^. Human fibroblast CCD-986sk was obtained from same distributor as other cell lines and maintained in Iscove’s Modified Dulbecco’s Medium (IMDM, SH30228.01; GE Healthcare, USA) with 10% FBS and 1% antibiotics. Cells were cultured at 37 ℃ in a humidified 5% CO_2_ incubator.

**Cellular toxicity assessments.** The cellular toxicity of the as-prepared nanoparticles was investigated from RAW 264.7 and U87-MG cell lines. Each cell line was cultured in a 12-well plate (~1.0 × 10^5^ cells per well) with a certain concentration of NF-SIONs. After incubating for 6, 12, 24 and 48 hours, the supernatant media was discarded and washed with 0.01 M PBS solution. Then, 1 mL of MTT (3-(4,5-dimethylthiazol-2-yl)-2,5-diphenyltetrazolium bromide, Sigma Aldrich) solution (0.5 mg·mL^-1^) was added to each well and the cells were incubated for an hour. Again, the supernatant solution was eliminated and 500 mL of DMSO was added to each well to break the cell membrane and dissolve the violet formazan crystals. Finally, the absorbance of each well was measured at 540 nm wavelength via microplate reader (mQuant, BioTek Instruments) and it was divided by that of the control experiment for relative comparison.

**Mouse models.** All animal experiments were carried out in accordance with the approved guidelines. All animal experimental protocols were approved by the Institutional Animal Care and Use Committee of Preclinical Research Institute in the Seoul National University Bundang Hospital (15099). 6-week-old male Balb/c nude mice were used in this preclinical experiment and those mice were purchased from Doo Yeol Biotech (Seoul, Korea). The animals were anesthetized with 2% isoflurane gas and 1 × 10^7^ U87-MG cells with cold PBS were injected into forelimb armpit of mice (n=3) subcutaneously with a sterile 26-gauge needle. After 2 weeks, we obtained U87-MG xenograft models for imaging experiment. To induce U87-MG glioblastoma orthotopic model, 5 × 10^4^ U87-MG-luc cells with cold PBS were prepared. Mice were anesthetized with an intraperitoneal injection of zoletil (20 mg·kg^-1^) and xylazine (10 mg·kg^-1^). Then U87-MG-luc cells were injected into the brain striatum through an entry point 0.5 mm anterior and 2 mm lateral to the bregma with Hamilton syringe (n=3) ^2^. Bioluminescence imaging (BLI) was conducted every three or four days for monitoring of tumor burden and growth on U87-MG glioblastoma orthotopic models. After 15 days, mice had substantial brain cancer at the point of imaging. All the animals had been administered on a regular diet and all experiments were performed in accordance with the guidelines by Institutional Animal Care and Use Committee (IACUC) and Seoul National University Animal Care.

**Immunofluorescence staining.** After euthanasia of glioblastoma orthotopic mouse models, brains were isolated and fixed by 4% paraformaldehyde in PBS, then were frozen with OCT compound at -80 ℃. Immunofluorescence staining method was utilized on 7 um-thick brain sections. All the slices were placed in 0.2% Tween 20 for 10 minutes and then incubated in 3% sodium deoxycholate solution on the shaker for 2-4 hours at room temperature. For blocking endogenous activity, 20-50% normal goat serum in 1% BSA-PBS solution was used and the slides were placed with this solution for 2 hours at 37 ℃. After that, staining of primary antibodies was conducted using GFAP (Ab5804, 1:200; Merck Millipore, Germany), CD11b (MCA711G, 1:200; Bio-Rad, USA), F4/80 (MF48000, 1:50; Life technologies, USA), CD86 (553689, 1:80; BD Biosciences, USA) and Iba1 (Ab5076, 1:400; Abcam, UK), then slices were incubated overnight at 4 ℃. Finally, they were washed and incubated with Alexa fluor 488 (A11034, 1:400; Life Technologies, USA) for GFAP and Alexa fluor 594 (A11007/A11037/A11058, 1:400; Life Technologies, USA) for CD11b, F4/80, CD86 and IbaI, and Hoechst (H3570, 1:750; Life Technologies, USA) serially. Each stained section was mounted with Gel/Mount™ (Mø 1; Biømeda Corporation, USA). The comparison of fluorescent images was observed with confocal microscope.

**References**

1. Sun, S. *et al.* Monodisperse MFe _2_ O _4_ (M = Fe, Co, Mn) Nanoparticles. *J. Am. Chem. Soc.* **126,** 273–279 (2004).

2. Kuroda, J. I. *et al.* Potent antitumor effect of SN-38-incorporating polymeric micelle, NK012, against malignant glioma. *Int. J. Cancer* **124,** 2505–2511 (2009).
